# The value of blood cytokines and chemokines in assessing COPD

**DOI:** 10.1186/s12931-017-0662-2

**Published:** 2017-10-24

**Authors:** Eric Bradford, Sean Jacobson, Jason Varasteh, Alejandro P. Comellas, Prescott Woodruff, Wanda O’Neal, Dawn L. DeMeo, Xingnan Li, Victor Kim, Michael Cho, Peter J. Castaldi, Craig Hersh, Edwin K. Silverman, James D. Crapo, Katerina Kechris, Russell P. Bowler

**Affiliations:** 1 Division of Pulmonary, Critical Care, and Sleep Medicine, Department of Medicine, National Jewish Health, 1400 Jackson St., K715, Denver, CO 80206 USA; 2 Division of Pulmonary Sciences and Critical Care Medicine, Department of Medicine, University of Colorado Denver, University of Colorado Anschutz Medical Campus, Research Building 2, 9th Floor, 12700 E. 19th Ave, Aurora, CO USA; 3 Department of Biostatistics and Informatics, University of Colorado Denver, Colorado School of Public Health, Mail Stop B119, 13001 E. 17th Place, Aurora, CO 80045 USA; 4 Channing Division of Network Medicine and the Division of Pulmonary and Critical Care Medicine, Department of Medicine, Brigham and Women’s Hospital, Harvard Medical School, Boston, MA 02115 USA; 5 Division of General Internal Medicine and Primary Care, Brigham and Women’s Hospital, Boston, Massachusetts USA; 6 University of Iowa, Internal Medicine, 200 Hawkins Dr C331-GH, Iowa City, IA 52242 USA; 7 UCSF, Division of Pulmonary and Critical Care Medicine and Cardiovascular Research Institute, Box 0130, Rm HSE 1305, 513 Parnassus Ave, San Francisco, CA 94143 USA; 8 Cystic Fibrosis/Pulmonary Research and Treatment Center, University of North Carolina at Chapel Hill, Chapel Hill, NC USA; 9 Department of Medicine, University of Arizona College of Medicine, Tucson, AZ USA; 10 Temple University School of Medicine, Pulmonary and Critical Care Medicine, 785 Parkinson Pavilion, 3401 North Broad Street, Philadelphia, PA 19140 USA; 11 Tufts Medical Center, ICRHPS, 800 Washington St, Box 63, Boston, MA 02111 USA

## Abstract

**Background:**

Blood biomarkers are increasingly used to stratify high risk chronic obstructive pulmonary disease (COPD) patients; however, there are fewer studies that have investigated multiple biomarkers and replicated in multiple large well-characterized cohorts of susceptible current and former smokers.

**Methods:**

We used two MSD multiplex panels to measure 9 cytokines and chemokines in 2123 subjects from COPDGene and 1117 subjects from SPIROMICS. These biomarkers included: interleukin (IL)-2, IL-6, IL-8, IL-10, tumor necrosis factor (TNF)-α, interferon (IFN)-γ, eotaxin/CCL-11, eotaxin-3/CCL-26, and thymus and activation-regulated chemokine (TARC)/CCL-17. Regression models adjusted for clinical covariates were used to determine which biomarkers were associated with the following COPD phenotypes: airflow obstruction (forced expiratory flow at 1 s (FEV_1_%) and FEV_1_/forced vital capacity (FEV_1_/FVC), chronic bronchitis, COPD exacerbations, and emphysema. Biomarker-genotype associations were assessed by genome-wide association of single nucleotide polymorphisms (SNPs).

**Results:**

Eotaxin and IL-6 were strongly associated with airflow obstruction and accounted for 3–5% of the measurement variance on top of clinical variables. IL-6 was associated with progressive airflow obstruction over 5 years and both IL-6 and IL-8 were associated with progressive emphysema over 5 years. None of the biomarkers were consistently associated with chronic bronchitis or COPD exacerbations. We identified one novel SNP (rs9302690 SNP) that was associated with CCL17 plasma measurements.

**Conclusion:**

When assessing smoking related pulmonary disease, biomarkers of inflammation such as IL-2, IL-6, IL-8, and eotaxin may add additional modest predictive value on top of clinical variables alone.

**Trial registration:**

COPDGene (ClinicalTrials.gov Identifier: NCT02445183).

Subpopulations and Intermediate Outcomes Measures in COPD Study (SPIROMICS) (ClinicalTrials.gov Identifier: NCT 01969344).

**Electronic supplementary material:**

The online version of this article (10.1186/s12931-017-0662-2) contains supplementary material, which is available to authorized users.

## Background

Chronic obstructive pulmonary disease (COPD) is typically caused by decades of exposure to smoke, dust or other inhaled toxins. The lung is the primary portal of exposure and bears most of the disease burden. Smoking related lung injury includes airflow obstruction, emphysema, chronic bronchitis, and lung cancer; however, there is also substantial evidence that tobacco smoke causes systemic disease. For instance, tobacco smoking is a major risk factor for cardiovascular disease as well as extrapulmonary malignancies such as bladder, stomach and pancreas [1]. Despite more than 50 years of knowledge that smoking causes both lung and systemic disease, the molecular basis for this is not fully understood. Furthermore, most smokers do not develop clinical lung disease such as COPD, emphysema, and chronic bronchitis and there is marked heterogeneity in disease manifestations in those who do. Since more than 100 million people in the United States and nearly 1 billion people worldwide are current or former smokers, there is a great need to identify diagnostic and prognostic biomarkers to assess disease risk and severity as well as to identify potential novel therapeutic targets.

Two strategies exist for developing biomarkers of COPD. First, one can obtain lung biosamples such as exhaled breath, sputum, bronchoalveolar lavage fluid (BALF), and lung brushes and biopsies. Exhaled breath is non-invasive, but has poor reproducibility and low protein content. Sputum requires expertise and time. BALF and lung brushes and biopsies may provide a more direct readout of the lung compartment; however, these sampling techniques are invasive, expensive, and have more than minimal risk. An alternative strategy for identifying COPD biomarkers is systemic biosampling, most commonly by obtaining plasma or serum and less commonly urine. The primary advantage of this strategy is ease in obtaining samples, low risk, and high reproducibility. The disadvantage is that blood may have a smaller biomarker signal compared to a sample obtained directly from the lung.

There are several blood biomarkers of varying value in predicting COPD affection status (case versus control), severity, and disease progression [2–5]. For instance, fibrinogen and C reactive protein (CRP), both non-specific markers of inflammation, tend to correlate with COPD severity and risk of exacerbations [6–21], although data are conflicting [22]. A protein which is abundantly expressed in the lung epithelium, the soluble receptor for advanced glycation end-products (sRAGE), is inversely correlated with emphysema and airflow obstruction [23–26]. Lung specific proteins such as surfactant protein D (SP-D) and club cell-16 (CC16) are also attractive COPD biomarkers. SP-D has been associated with COPD [14, 27–29], and emphysema [25] and possibly exacerbation frequency [16, 29]. CC16 may correlate with airflow obstruction [30] and emphysema [25]. The major limitation to many of the previous publications include: small sample size, limited clinical phenotyping, and lack of reproducibility in an independent cohort. In this study, we address some of these limitations by studying 9 blood chemokines and cytokines in more than 3000 subjects from two well phenotyped longitudinal cohorts of smokers: COPDGene and SPIROMICS.

## Methods

### Study populations

This study includes two independent NIH-funded cohorts: COPDGene (ClinicalTrials.gov Identifier: NCT02445183) and Subpopulations and Intermediate Outcomes Measures in COPD Study (SPIROMICS) (ClinicalTrials.gov Identifier: NCT 01969344). The institutional review board at all participating sites approved the study protocols (Additional file 1: Table S1). Study participants provided written informed consent.

COPDGene is a multicenter prospective observational study funded by the NIH which enrolled 10,300 subjects 45–80 years old, with at least a 10 pack-year history of smoking, and who had not had an exacerbation of COPD for at least the previous 30 days. The cohort also includes 108 subjects who never smoked (< 100 lifetime cigarettes). Subjects were recruited from 2008 to 2011 and were invited to return for a 5-year follow up visit from 2013 to 2017. Blood was drawn into a vacutainer EDTA plasma tube, immediately spun, aliquoted, and frozen. The subset for this current analysis includes the first 2122 who returned and provided a blood sample during their 5-year follow up visit. Biomarker measurements were made using plasma from the 5-year follow up visit. Additional information on the COPDGene study and the collection of clinical, radiographic, and spirometry data has been described previously [31].

*SPIROMICS* is an ongoing multicenter prospective observational study funded by the NIH [17] that enrolled 2982 subjects between November 2011 and January 2015. Subjects were 40–80 years old at the time of enrollment. Subjects were categorized as non-tobacco smokers (< 1 pack-year; stratum 1) or smokers (> 20 pack-years; Stratum 2–4). At the baseline visit blood was drawn into a vacutainer EDTA plasma tube, immediately spun, aliquoted, and frozen. The subset for this current analysis was 1026 subjects with baseline blood samples including all subjects with history of smoking but no airflow obstruction (*N* = 551) and a random sample of those with COPD (*N* = 566). Additional information on the SPIROMICS study and the collection of clinical, radiographic, and spirometry data has been described previously [32].

### Clinical phenotype definitions

COPD was defined by post-bronchodilator forced expiratory volume in the first second (FEV_1_) to forced vital capacity (FVC) ratio of < 0.70. Smoker controls were current or former smokers without evidence of airflow obstruction (FEV_1_/FVC ≥ 0.70). Emphysema was defined by the percent of voxels with Hounsfield Units (HU) < −950 (%LAA) on inspiratory CT. Emphysema progression was defined as change in lung density adjusted for predicted total lung capacity (adj. g/L), but only available in the COPDGene cohort. Chronic bronchitis (CB) was defined as the subject reporting chronic cough and sputum production for at least 3 months per year for two consecutive years [33]. Moderate exacerbations were defined as those treated with steroids and/or antibiotics; severe exacerbations were defined as those resulting in hospitalization. For cross sectional analysis, subjects where further subcategorized as emphysema (LAA > 5%) or no emphysema (LAA ≤ 5%).

### Biomarker selection and measurement

In a previous COPDGene and SPIROMCIS study we used a 13-panel luminex-RBM assays to measure 114 candidate plasma and serum biomarkers [34]. Twenty-six of the biomarkers had more than 50% of the values below lower limit of detection (LLOD) and were not analyzed. From this list, we selected plasma biomarkers for further study on a different a Meso Scale Discovery (MSD, Rockville, Maryland) platform. Biomarker selection was based on these criteria: (1) inflammation chemokine or cytokine with plausible association with COPD-related phenotypes; (2) below lower limit of detection from a previous study using a luminex-RBM pane in COPDGene and SPIROMCIS subjects [34]; (3) had the majority of measurements within the limit of detection in a pilot project (*N* = 40) using a MSD V-PLEX Human Cytokine 30-Plex Kit. The 9 cytokines and chemokines that met these criteria and were run using two separate multiplex assays: assay 1 (cytokines)- interleukin (IL)-2, IL-6, IL-8, IL-10, tumor necrosis factor (TNF)-α, interferon (IFN)-γ assay 2 (chemokines)- eotaxin/CCL-11, eotaxin-3/CCL-26, and thymus and activation-regulated chemokine (TARC)/CCL-17. To determine assay coefficients of variation (CVs), first 200 cytokine assays and the first 240 chemokine assays were performed in duplicate. Assay characteristics of the MSD assays are shown in Additional file 1: Table S1. Values below the LLOD were assigned half the LLOD and values above the upper limit of detection (ULOD) were assigned the ULOD.

### Statistical analysis

Data sets used for analysis from COPDGene included: the COPDGene Phase 2 5000 data set from September 24, 2016. Data sets used for analysis from SPIROMICS included: the Core 4 datasets. R (v 3.2.0) was used for analysis unless otherwise indicated. Differences in demographic characteristics of study subjects were assessed using a t test or Chi squared test. Because of non-normality, biomarker values were log10 transformed (Additional file 1: Figure S1) and all statistical analysis was done with the log10 value of the biomarker. Statistical models and covariates were selected based on previous literature [9, 10, 14, 16, 25, 35, 36] as indicated in Additional file 1: Table S2. Akaike Information Criteria (AIC) was used to determine how well a model fit. The R^2^ (adj) reported refers to the percent variation of the phenotype explained by the biomarkers(s) over clinical covariates alone. The adjusted R^2^ (adj) was used for estimating the percent variation of FEV_1_% explained by the biomarkers over clinical covariates alone using the Core R package. For FEV_1_/FVC we reported the McFadden pseudo-R2 [37] using the betareg package. For chronic bronchitis we report the Cragg-Uhler pseudo R^2^ [38] using the pscl package. For decline in FEV_1_ and emphysema progression we report the marginal R^2^ [39] using the MuMin package. Biomarker(s) were considered to improve the model if the AIC was lower than clinical covariates alone and the *p*-value for the complete model was less than 0.05. *P*-values were combined using Stouffer’s Z-score method. Single nucleotide polymorphism (SNP)-biomarker associations were assessed in non-Hispanic White subjects with PLINK using genetic ancestry principal components, sex, age, body mass index (BMI), smoking pack years and current smoker status as previously described [34]. A cutoff of *P* < 10^−9^ was used to account for multiple biomarker testing. For subgroup analysis (Additional files 2 and 3), we calculated the *P* values for the individual cytokine associations in the same models that included the covariates described above. Significant P-values (*P* < 0.05) for the cytokine ß estimate in each clinical phenotype regression were shaded on a heatmap according to the -1og10 scale of the P – value. Colors were blue for negative associations, red for positive associations, and grey for insufficient endpoints.

## Results

### Demographics characteristics of subjects and associations with biomarkers

Baseline characteristics of the COPDGene and SPIROMICS subjects are shown in Table 1. The COPDGene subjects included in this study were generally similar to the SPIROMICS subjects, but the SPIROMICS subjects were slightly younger, had lower BMI, greater smoking intensity, and included a lower percentage of subjects with moderate COPD and a higher percentage of subjects with severe COPD. Most of the cytokines and chemokines were strongly associated with smoking status and also showed association with age, race, BMI, and gender (Additional file 1: Tables S3–S6). For instance, current smoking was associated with lower IL-2 in both cohorts, but higher CCL17 (TARC). Because of these associations, these covariates were included in statistical models. Cytokines were also associated with multiple different complete blood cell counts consistently between cohorts (Additional file 1: Figure S2).

**Table 1:** Demographics of subjects

	Never smokers *N* = 25	COPDGene *N* = 2098	SPIROMICS *N* = 1117	p-value (SPIROMICS vs COPDGene) (current and former smokers)
Age (years)	57.5 (7)	65.8 (8.9)	62.7 (9)	< 10^−04^
Gender Male	36.0%	50.9%	51.9%	0.6931
Race				
White	100.0%	71.2%	74.9%	
Black	0.0%	28.4%	20.8%	
Other	0.0%	0.0%	4.3%	
Current Smoker	0%	34.7%	40.5%	0.0009
BMI (kg/m^2^)	25.7 (4.2)	28.8 (6.3)	28.2 (5.4)	0.0028
ATS Smoking, pack-years	0 (0)	45.1 (24)	47.6 (24.7)	0.0054
FEV_1_ (% predicted)	105.5 (12)	76.1 (25.6)	76.1 (28.7)	0.9906
FEV_1_/FVC	0.8 (0)	0.7 (0.2)	0.6 (0.2)	< 10^−04^
BODE Index	0.2 (0.4)	1.3 (1.7)	1.5 (2)	0.0084
SGRQ	2.7 (3.5)	23.6 (21.1)	32 (21.2)	< 10^−04^
Emphysema (% LAA < −950 HU)	1 (1.3)	6.5 (10.2)	7.3 (10.4)	0.0386
Chronic Bronchitis (%)	4%	15.2%	20.9%	< 10^−04^
Duration of participation in study (years)	NA	6.7 (1.2)	2.2 (0.9)	< 10^−04^
Decline in FEV_1_ (ml/year)	NA	−38.1 (53.4)	−48.5 (144.1)	0.0416
Exacerbations (#/year)	0 (0)	0.3 (0.8)	0.5 (1.0)	< 10^−04^
Never Smoker	100%	0%	0%	
Spirometry category				
PRISm	0%	10.4%	0%	
Control Smoker	0%	41.5%	49.3%	
GOLD 1	0%	9.3%	7.9%	
GOLD 2	0%	20.4%	17.5%	
GOLD 3	0%	12.2%	18.1%	
GOLD 4	0%	5.8%	7.1%	

*Abbreviations*: *SGRQ* St. George’s Respiratory Questionnaire, *HU* Hounsfield Unites, *BODE* Body-mass index, airflow Obstruction, Dyspnea, and Exercise, *PRISm* preserved ratio, impaired spirometry, *GOLD* Global Initiative for Chronic Obstructive Lung Disease

*P*-values are not applicable to race, ever smoking, and spirometry category because these criteria were used as inclusion criteria in one or both studies

### Biomarkers associated with COPD affection status and airflow obstruction

**Fig. 1:**
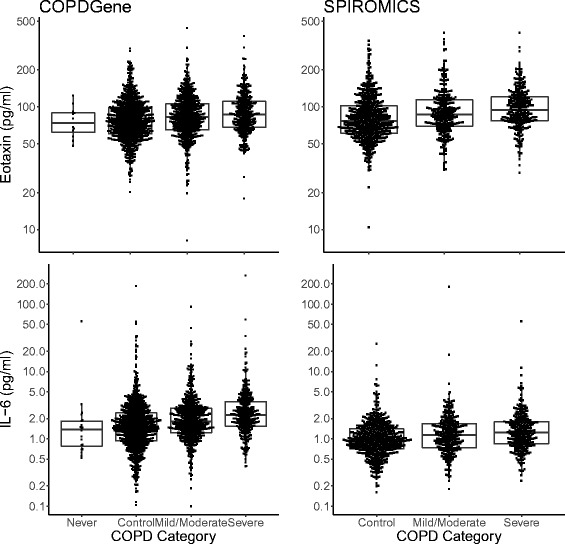
Plasma eotaxin and IL-6 are higher in subjects with COPD. Never smokers (never) and current and former smokers with no COPD (control), mild/moderate COPD (Mild/Moderate), or severe or very severe COPD (Severe)

Four biomarkers (eotaxin, IL-6, IL-8, and IL-10) were independently associated (*P* < 0.05) with worse airflow obstruction (FEV_1_%) in both cohorts, even after adjustment for clinical covariates (Table 2). Similar associations were seen for FEV_1_/FVC (Additional file 1: Table S7). Both eotaxin and IL-6 were significantly higher in cases compared to controls and were higher in severe COPD compared to mild/moderate COPD (Fig. 1). In a full regression model with clinical covariates, plasma IL-6 accounted for an additional 4–5% of variance of FEV_1_% and 2–3% variance of FEV_1_/FVC. Other biomarkers accounted for less of the variance in these and other outcomes (Additional file 1: Table S8). Similar results were seen in subgroup analyses when subjects were grouped on presence or absence of airflow obstruction (GOLD 1–4), chronic bronchitis, and emphysema (Additional file 2); however, dividing the cohort into 4 or more subgroups substantially reduced the power of the analyses. When adding biomarkers to a model that included clinical covariates, higher IL-6 was also associated with more rapid progression of airflow obstruction at 5 years in the COPDGene cohort, but not over a 1 year follow up in SPIROMICS (Additional file 1: Table S9). When stratifying the COPDGene subjects by GOLD groups, higher IL-6 was still associated with more rapid decline, but the association was no longer significant when clinical covariates were included in the model (Additional file 1: Table S10). In the COPDGene cohort, there was a significant association with 5-year decline in FEV_1_ and IL-6 in subjects who did not have COPD or emphysema and significant association with 5-year decline in FEV_1_ and IL-8 in subjects who had chronic bronchitis, but no emphysema (Additional file 3). The amount of additional variance in progression of FEV_1_ decline explained by a IL-6 in addition to clinical covariates was 3%.

**Table 2:** Biomarkers associated with FEV_1_%

	COPDGene			SPIROMICS			Combined
Biomarker	ß	R^2^_(adj)_	P	ß	R^2^_(adj)_	P	P
Eotaxin	−12.2	0.007	0.0001	−31.3	0.039	< 10^−10^	< 10^−12^
CCL26	−1.7	0.000	0.2225	−3.4	0.001	0.1095	0.0679
CCL17	0.1	0.000	0.9383	−3.7	0.001	0.1664	0.3525
IFN-γ	−6.2	0.006	0.0003	−4.5	0.002	0.0881	0.0002
IL10	−4.1	0.003	0.0072	−6.2	0.003	0.0442	0.0013
IL2	−2.8	0.002	0.0426	−7.2	0.007	0.0043	0.0016
IL6	−16.2	0.049	< 10^−23^	−21.1	0.042	< 10^−11^	< 10^−34^
IL8	−4.2	0.002	0.0226	−9.9	0.005	0.0091	0.0008
TNF-α	−7.0	0.006	0.0004	−6.4	0.001	0.1725	0.0003

R^2^_(adj)_ is the partial amount of variance explained by the biomarker in models with clinical covariates

### Biomarkers associated with emphysema severity and progression, chronic bronchitis and COPD exacerbations

Although none of the 9 biomarkers had independent cross sectional associations with emphysema severity at a single time point (LAA% < −950 HU), higher IL-6 and IL-8 were associated with progression of CT assessed emphysema over 5 years (Table 3). The IL-6 association with emphysema progression was also seen in subgroup analysis which included only subjects without COPD or chronic bronchitis and no emphysema at baseline (Additional file 3).

**Table 3:** biomarkers associated with progression of emphysema

	COPDGene		
Biomarker	Value	s.e.	P
Eotaxin	0.177	0.102	0.0835
CCL26	−0.011	0.046	0.8060
CCL17	0.010	0.048	0.8390
IFN-γ	−0.039	0.056	0.4865
IL10	0.081	0.050	0.1093
IL2	−0.033	0.047	0.4857
IL6	0.153	0.054	0.0042
IL8	0.145	0.062	0.0196
TNF-α	0.088	0.066	0.1832

### Biomarkers associated with emphysema severity and progression, chronic bronchitis and COPD exacerbations

**Fig. 2:**
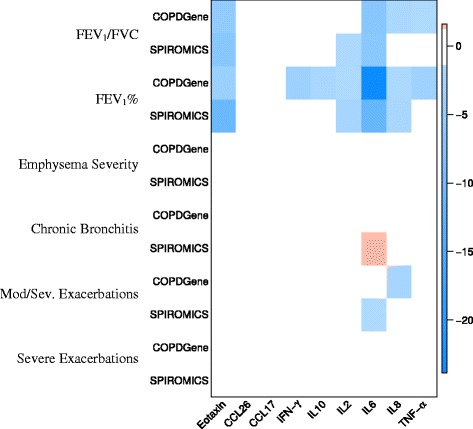
Heat map showing associations between cytokines and chemokines and COPD clinical phenotypes in the COPDGene and SPIROMICS cohorts. The intensity of the color represents the log of the *P*-value with *red* indicating positive associations and *blue* indicating negative associations

None of the 9 biomarkers were reproducibly associated with chronic bronchitis. Although there were other chemokines and cytokines that were associated with exacerbations in either COPDGene or SPIROMICS (Fig. 2), none of these associations were significant in both cohorts.

### Relationship between genotype and biomarker level

Because we recently reported that genetic factors can also influence many different biomarker measurements [34], we assessed associations between genetic variants and biomarker measurements using protein quantitative trait locus (pQTL) analysis (Additional file 1: Figure S3). The rs9302690 SNP in *CCL17* was the only genetic locus significantly associated with a biomarker measurement in both cohorts (*P* < 10^−11^ in COPDGene and *P* < 10^−10^ in SPIROMICS). The minor allele was (A) was associated with higher levels of CCL17 (Additional file 1: Figure S4) and occurs in intron 3 of *CCL17*. This is the first report of this SNP being association with CCL17.

## Discussion

Although tobacco smoke is inhaled though the lung, there is substantial evidence that tobacco exposure has systemic manifestations and is associated with extra-pulmonary disease [2–5]. While the mechanisms of tobacco smoke-induced systemic injury are not fully understood, inflammation is thought to play a key role. This study is one of largest multiplex investigations of cytokines and chemokine biomarkers to date and is one of the few that includes two large, independent, well phenotyped cohorts of current and former smokers. Although we found that most of the cytokines and chemokines were associated with some COPD phenotypes, only eotaxin and IL-6 were strongly and consistently associated with airflow flow limitation in both cohorts, even after adjustment for important clinical covariates. None of the nine biomarkers were associated with chronic bronchitis. None of the 9 biomarkers were consistently associated with COPD exacerbations, which is similar to what has been recently reported for COPD exacerbations in a more extensive study of other blood biomarkers, albeit with few subjects [40].

Eotaxin-1 (CCL11) is a potent eosinophil chemoattractant that is normally associated with asthma [41], but is known to play a role in other mucosal diseases such as inflammatory bowel disease (see review [42]). Eotaxin and eotaxin receptor (CCR3) positive cells are higher in acute exacerbations of chronic bronchitis as well as asthma [43]; however, the published associations between plasma eotaxin and COPD in non-exacerbating subjects are contradictory, possibly because most include only a small number of subjects. For instance, in 50 FORTE study participants (34 stable and 16 rapid decliners) and 11 controls, plasma eotaxin-1 was lower in rapid decliners compared to stable COPD patients, but eotaxin was also significantly lower in stable COPD subjects compared to normal controls (*p* < 0.03) [44]. In a different study of 21 COPD subjects and 9 controls, eotaxin was higher in COPD patients compared to controls [45]. In our study, which included more than 3000 subjects, eotaxin was higher in COPD subjects in both cohorts compared to control subjects with no COPD and a comparable smoking history. Eotaxin was higher in subjects with chronic bronchitis and was positively associated with neutrophils and negatively associated with eosinophil counts. These findings suggest that eotaxin is associated with a neutrophilic/inflammatory COPD, but does not appear to be independently associated exacerbations or higher eosinophils, as might be expected with asthma exacerbations.

Another strong association was between IL-6 and COPD affection status, airflow limitation and emphysema progression. IL-6 is a 26 kDa, 184 amino acid multifunctional glycoprotein and pro-inflammatory cytokine that is produced in a variety of stromal and immune cells and which is associated with a large number of pulmonary and extra-pulmonary inflammatory diseases (see reviews [46, 47]). In this study, which is appreciably larger than previously published studies, we found that IL-6 was associated with both case-control status, COPD severity, rate of decline in spirometry, and independently associated with emphysema progression as assessed by CT scans; however, it was not independently associated with exacerbations. The case-control associations are consistent with several large population studies. For example, in the Health, Aging, and Body Composition study which included 3075 subjects [48], the Framingham Heart Study which included 2553 subjects [49], the Rotterdam Study which included 572 older subjects [50], plasma IL-6 was higher in those with COPD compared to those without. This is consistent with a recent meta-analysis of IL-6 and COPD, which included 1891 COPD subjects and 4946 controls from 33 studies [51]. This meta-analysis also reported a non-statistically significant trend toward the mild-moderate COPD subjects having lower plasma IL-6 compared to severe COPD subjects; however, IL-6 was not associated with disease severity in 1793 subjects from in the ECLIPSE, which primarily included COPD subjects [14]. IL-6 was also not associated with decline in the ECLIPSE cohort. Since IL-6 was strongly associated with neutrophils in both cohorts, this would suggest that IL-6 may drive the inflammatory phenotype which promotes progressive airflow limitation. While our analysis showed a statistically significant independent association with decline in lung function, adding IL-6 to the model with clinical covariates (e.g. low FEV_1_%) added only about 4–5% to the explanation of variance. This is consistent with the concept that subjects with low lung function have an inflammatory phenotype and are predisposed to more rapid decline in lung function, and that adding biomarkers to these prediction models will add a small, but additional benefit to predicting decline on top of clinical covariates.

The COPDGene study is one of the largest current and former smoker cohorts with long term CT follow up and this study is one of the first to report IL-6 as an independent biomarker of emphysema progression. A pathologic role for IL-6 is supported by several observations. First, IL-6 binds to IL-6 receptor and signals through at gp130 subunit; it transduces inflammatory gene transcription through JAK-STAT pathways. Second, genetic blockade of the IL-6 receptor subunit gp130 blocks cigarette smoke induced emphysema [52]. Third, IL-6 is associated with cardiovascular disease in COPD patients [53] and recent literature supports a vascular etiology of emphysema [54]. Although IL-6 specific treatments (e.g. tocilizumab) have been developed, but not yet tried as a treatment for COPD, one case report describes worsening of emphysema during treatment for rheumatoid arthritis [55]. Thus, anti-IL-6 treatment in COPD should be done with caution.

In additional to eotaxin and IL-6, IL-2, IL-8, and IL-10 were also found to be elevated in COPD patients, although they accounted for only a small amount of the variance in airflow obstruction compared to IL-6 and eotaxin. For several of these cytokines, there are only smaller studies previously published. In a study of 10 COPD patients and 10 controls, ex vivo IL-2 release from stimulated T-cells was higher in COPD patients compared to smoking controls [56]. In the 50 FORTE study participants discussed above, IL-2 was higher in COPD patients, but was lower in rapid decliners compared to stable COPD patients [44]. Similarly, in small studies IL-8 has been reported to be elevated in COPD patients in smaller studies with less than 100 subjects [57, 58]. This is the first large study to show that IL-8 is independently associated with progression of emphysema by CT scan and additional studies in independent longitudinal COPD cohorts should consider measuring IL-8. Similarly, we find that IL-10 is associated with worse COPD; however, there are only a few published studies, which may be underpowered to confirm or refute these observations. For example in a study of 94 COPD patients and 45 controls, IL-10 was no different between COPD patients and controls, but lower than in healthy non-smokers [59]. Since IL-10 was not associated with progression of COPD or emphysema, it is unclear whether it may be a useful predictive marker.

Although CCL17 is more expressed in airway cells from COPD patients and plays a role in Th2 inflammation [60], we found no association with any COPD phenotypes. However, our study is the first report of the rs9302690 SNP being a pQTL for CCL17, with the minor allele being associated with higher plasma levels of CCL17. This finding may be relevant to other clinical investigators because *CCL17* is expressed in many tissues and has been associated with atopic dermatitis [61] and Hodgkin’s Lymphoma [62]. In GTex analysis, the rs9302690 SNP is also a gene expression QTL (eQTL) (GTEx V6p) with the minor allele being associated with higher CCL mRNA in esophagus and testes and lower expression in adrenal and pituitary tissue. Thus, both CCL17 gene and protein expression should be adjusted for the rs9302690 genotype.

While this study was unique in that it featured two large well-characterized cohorts, confirmed strong associations of IL-6 and eotaxin, identified new pQTL SNPs, and identified potentially new biomarkers of COPD and emphysema progression, there were some important limitations. Most importantly, biomarkers were assessed at only a single time point and thus one cannot determine whether the biomarkers temporally fluctuate with disease activity. We also only studied 9 biologically plausible biomarkers, but there are new platforms which will permit the simultaneous measurements of hundreds or thousands of proteins, even if these platforms may not be designed to assay low abundant proteins such as interleukins. Also, although subgrouping into phenotypes showed that some cytokines such as IL-6 were associated with severity and progression of airflow obstruction and emphysema even in subjects without COPD or emphysema at baseline, other subgroup analyses were limited by the loss of power that occurred when subgroup sizes dropped below 500 subjects. This might suggest that biomarkers might be useful markers of disease progression in current and former smokers who do not yet manifest COPD or emphysema. Finally, other limitations of this study include the relatively low number of nonsmokers and only limited progression data in one of the cohorts (SPIROMICS).

## Conclusion

In summary, we show that selected cytokines such as eotaxin and IL-6 explain a moderate amount of the clinical COPD phenotypic variance (3–5%) when added to models with clinical covariates. Eotaxin, IL-6, and IL-8 may also have some value independent of clinical variables in predicting progression, although this should be demonstrated in other long term longitudinal cohorts besides COPDGene. We remain optimistic that some of these biomarkers may be useful for clinical trials, in which biomarkers might define inclusion criteria in order to limit trials to a subgroup of patients, e.g., those more likely to progress and therefore more likely to benefit from a given intervention. This has the potential to lead to the identification of a therapies from which a specific group of patients may benefit. In addition, biomarker combinations may serve as surrogate endpoints if they are prospectively demonstrated to correlate with clinically relevant outcomes. For these reasons, consideration should be given to development of panels of multiple biomarkers for COPD observational and interventional studies.

## Additional files

Additional file 1: Table S1. Range of Cytokines and Chemokines for MSD assay. **Table S2.** Regression Models and Covariates for each Phenotype. **Table S3.** Biomarkers associated with age. **Table S4.** Biomarkers associated with female gender. **Table S5.** Biomarkers associated with BMI. **Table S6.** Biomarkers associated with current smoking. **Table S7.** Biomarkers associated with FEV_1_/FVC. **Table S8.** Amount of variance explained by biomarker and clinical covariate alone and in combination. **Table S9.** Biomarkers associated with decline in FEV_1_ (ml/yr) for all subjects. **Table S10.** Biomarkers associated with decline in FEV_1_ (ml/yr) by COPD or no COPD in COPDGene subjects. **Figure S1.** Subtyping of subjects based on airflow obstruction (FEV_1_/FVC) and emphysema severity (LAA% < −950 HU). The vertical line represents the cutoff for COPD (post-bronchodilator FEV_1_/FVC < 0.7). The horizontal line represents the cutoff for emphysema (LAA > 5%). Subjects with chronic bronchitis are shown by red cross and those without a blue circle. The upper panel shows those included in analysis and the lower panels show the whole cohort for COPDGene (left) and SPIROMICS (right). **Figure S2.** Histograms of chemokines and cytokines in COPDGene and SPIROMICS (log10 transformed). Units are pg/ml. **Figure S3.** Pearson correlations between cytokines/chemokines and cell counts in COPDGene and SPIROMICS. Red squares are positive correlation coefficients and blue negative correlation coefficients with *P* < 0.05. Cell counts were obtained by automated complete blood cell counts. The shading of each cell represents the correlation coefficient as indicated in the legend. **Figure S4.** Combined Manhattan plots for all 9 biomarker-genotype associations in non-Hispanic White subjects from the COPDGene cohort \. The redline represents genome wide significance level adjusted for multiple testing (*P* < 10^−9^). Results for all 9 biomarkers are superimposed on the graph. Only one SNP was significantly associated with a biomarker (rs9302690 in CCL17; *P* = 10^−11^). (DOCX 11977 kb)

Additional file 2: Cross Sectional Associations by Subgroup Heat Map. (PDF 156 kb)

Additional file 3: Associations with Disease Progression by Subgroup Heatmap. (PDF 7 kb)
