# Supplementary material for: The value of blood cytokines and chemokines in assessing COPD
Source: Respir Res. 2017 Oct 24;18:180. doi: 10.1186/s12931-017-0662-2 (PMC5655820; doi:10.1186/s12931-017-0662-2)
Supplement: Supplementary file 1 — Range of Cytokines and Chemokines for MSD assay. Table S2. Regression Models and Covariates for each Phenotype. Table S3. Biomarkers associated with age. Table S4. Biomarkers associated with female gender. Table S5. Biomarkers associated with BMI. Table S6. Biomarkers associated with current smoking. Table S7. Biomarkers associated with FEV1/FVC. Table S8. Amount of variance explained by biomarker and clinical covariate alone and in combination. Table S9. Biomarkers associated with decline in FEV1 (ml/yr) for all subjects. Table S10. Biomarkers associated with decline in FEV1 (ml/yr) by COPD or no COPD in COPDGene subjects. Figure S1. Subtyping of subjects based on airflow obstruction (FEV1/FVC) and emphysema severity (LAA% < −950 HU). The vertical line represents the cutoff for COPD (post-bronchodilator FEV1/FVC < 0.7). The horizontal line represents the cutoff for emphysema (LAA > 5%). Subjects with chronic bronchitis are shown by red cross and those without a blue circle. The upper panel shows those included in analysis and the lower panels show the whole cohort for COPDGene (left) and SPIROMICS (right). Figure S2. Histograms of chemokines and cytokines in COPDGene and SPIROMICS (log10 transformed). Units are pg/ml. Figure S3. Pearson correlations between cytokines/chemokines and cell counts in COPDGene and SPIROMICS. Red squares are positive correlation coefficients and blue negative correlation coefficients with P < 0.05. Cell counts were obtained by automated complete blood cell counts. The shading of each cell represents the correlation coefficient as indicated in the legend. Figure S4. Combined Manhattan plots for all 9 biomarker-genotype associations in non-Hispanic White subjects from the COPDGene cohort \. The redline represents genome wide significance level adjusted for multiple testing (P < 10−9). Results for all 9 biomarkers are superimposed on the graph. Only one SNP was significantly associated with a biomarker (rs9302690 in CCL17; P = 10−11). (DOCX [file 12931_2017_662_MOESM1_ESM.docx]

|  | | | Table S1; Range of Cytokines and Chemokines for MSD assay | | | | | | | |  |
| --- | --- | --- | --- | --- | --- | --- | --- | --- | --- | --- | --- |
|  | |  |  | |  | COPDGene | | | SPIROMICS | | |
| Full name | | abbreviation | limit of detection (pg/mL) | | CV% median (IQR) | # below LOD | Median value | # above LOD | # below LOD | Median value | # above LOD |
| Interleukin-2 | | IL-2 | 0.09–938 | | 7 (3 – 12) | 311 | 0.18 | 0 | 84 | 0.27 | 0 |
| Interleukin-6 | | IL-6 | 0.06–488 | | 5 (2 – 8) | 1 | 1.78 | 1 | 0 | 1.06 | 0 |
| Interleukin-8 | | IL-8 | 0.04–375 | | 4 (1 – 5) | 0 | 13.53 | 1 | 0 | 5.16 | 0 |
| Interleukin-10 | | IL-10 | 0.03–233 | | 5 (2 – 9) | 0 | 1.31 | 0 | 0 | 0.36 | 0 |
| tumor necrosis factor-α | | TNFα | 0.05–458 | | 5 (2 – 8) | 0 | 10.04 | 0 | 0 | 2.29 | 0 |
| Interferon gamma | | IFNγ | 0.20–938 | | 4 (2 – 8) | 1 | 4.11 | 0 | 0 | 4.61 | 1 |
| Eotaxin | | eotaxin | 3.26–1120 | | 10 (3-33) | 2 | 80.03 | 0 | 0 | 83.43 | 0 |
| C-C motif chemokine 17 | | CCL17 | 0.22–1120 | | 16 (8-30) | 1 | 52.34 | 10 | 0 | 56.95 | 0 |
| C-C motif chemokine 26 | | CCL26 | 1.77–3750 | | 11 (3-25) | 11 | 8.38 | 6 | 3 | 10.38 | 0 |

| Table S2: Regression Models and Covariates for each Phenotype | | | | | | | | | | | | | | | | |
| --- | --- | --- | --- | --- | --- | --- | --- | --- | --- | --- | --- | --- | --- | --- | --- | --- |
| Phenotype | Outcome | | Regression model | Age | Gender | Race | Smoking  (current or former) | ATS Pack Years | Height | Height^2^ | Age^2^ | FEV_1_% | BMI | Previous COPD Exacerbations | SGRQ | CT scanner |
| Spirometry | FEV_1_% | | Linear |  |  |  |  |  |  |  |  |  |  |  |  |  |
|  | FEV_1_/FVC | | Linear | **X** | **X** |  |  |  |  |  |  |  |  |  |  |  |
|  | FEV1 ml/yr | | Mixed | **X** | **X** |  | **X** | **X** |  | **X** | **X** | **X** |  |  |  |  |
| CT | Emphysema severity (LAA%) | | Beta |  |  |  |  |  |  |  |  |  | **X** |  |  | **X** |
|  | Emphysema progression (adjusted lung density) | | Mixed | **X** | **X** |  |  | **X** |  | **X** | **X** | **X** | **X** | **X** | **X** |  |
| Clinical | Chronic Bronchitis | | logistic | **X** | **X** |  |  | **X** | **X** |  |  |  |  | **X** |  |  |
|  | Exacerbations | | Negative binomial with zero inflation |  | **X** |  |  |  |  |  |  | **X** |  | **X** | **X** |  |
|  | |  | | | | | | | | | | | | | | |

| Table S3: Biomarkers associated with age |  |  |  |  |  | |
| --- | --- | --- | --- | --- | --- | --- |
|  |  | COPDGene |  |  | SPIROMICS | |
| biomarker | ß | sd | P | ß | sd | P |
| Eotaxin | 0.002 | 0.000 | < 10^-7^ | 0.003 | 0.001 | < 10^-5^ |
| CCL26 | 0.000 | 0.001 | 0.8234 | 0.000 | 0.001 | 0.8558 |
| CCL17 | -0.001 | 0.001 | 0.2967 | 0.000 | 0.001 | 0.6724 |
| IFN-γ | 0.003 | 0.001 | 0.0001 | 0.004 | 0.001 | 0.0005 |
| IL10 | 0.000 | 0.001 | 0.8799 | 0.002 | 0.001 | 0.0894 |
| IL2 | 0.005 | 0.001 | < 10^-4^ | 0.003 | 0.001 | 0.0036 |
| IL6 | 0.003 | 0.001 | 0.0006 | 0.003 | 0.001 | 0.0003 |
| IL8 | 0.002 | 0.001 | 0.0160 | 0.003 | 0.001 | 0.0005 |
| TNF-α | 0.002 | 0.001 | 0.0107 | 0.004 | 0.001 | < 10^-9^ |

| Table S4: Biomarkers associated with female gender | | | | | | |
| --- | --- | --- | --- | --- | --- | --- |
|  |  | COPDGene |  |  | SPIROMICS | |
| biomarker | ß | sd | P | ß | sd | P |
| Eotaxin | -0.025 | 0.008 | 0.0014 | -0.023 | 0.011 | 0.0387 |
| CCL26 | -0.088 | 0.017 | < 10^-6^ | -0.128 | 0.025 | < 10^-6^ |
| CCL17 | -0.019 | 0.017 | 0.2748 | -0.015 | 0.019 | 0.4375 |
| IFN-γ | 0.029 | 0.014 | 0.0459 | 0.023 | 0.020 | 0.2456 |
| IL10 | -0.035 | 0.016 | 0.0304 | -0.031 | 0.017 | 0.0678 |
| IL2 | -0.044 | 0.019 | 0.0206 | -0.070 | 0.021 | 0.0008 |
| IL6 | -0.004 | 0.015 | 0.7692 | -0.019 | 0.017 | 0.2706 |
| IL8 | 0.001 | 0.013 | 0.9661 | -0.004 | 0.014 | 0.7949 |
| TNF-α | 0.007 | 0.012 | 0.5776 | 0.012 | 0.011 | 0.2638 |

| Table S5: Biomarkers associated with BMI |  |  |  |  |  | |
| --- | --- | --- | --- | --- | --- | --- |
|  |  | COPDGene |  |  | SPIROMICS | |
| biomarker | ß | sd | P | ß | sd | P |
| Eotaxin | -0.005 | 0.001 | < 10^-14^ | -0.006 | 0.001 | < 10^-18^ |
| CCL26 | 0.003 | 0.001 | 0.0377 | 0.001 | 0.002 | 0.5882 |
| CCL17 | -0.002 | 0.001 | 0.0791 | -0.004 | 0.002 | 0.0252 |
| IFN-γ | -0.001 | 0.001 | 0.2626 | 0.003 | 0.002 | 0.1345 |
| IL10 | -0.003 | 0.001 | 0.0418 | 0.000 | 0.002 | 0.8097 |
| IL2 | -0.002 | 0.002 | 0.1026 | 0.003 | 0.002 | 0.1636 |
| IL6 | 0.008 | 0.001 | < 10^-6^ | 0.007 | 0.002 | < 10^-5^ |
| IL8 | -0.003 | 0.001 | 0.0018 | -0.002 | 0.001 | 0.1485 |
| TNF-α | 0.001 | 0.001 | 0.4663 | 0.004 | 0.001 | 0.0001 |

| Table S6: Biomarkers associated with current smoking |  |  |  |  |  | |
| --- | --- | --- | --- | --- | --- | --- |
|  |  | COPDGene |  |  | SPIROMICS | |
| biomarker | ß | sd | P | ß | sd | P |
| Eotaxin | 0.014 | 0.008 | 0.0853 | 0.028 | 0.011 | 0.0108 |
| CCL26 | -0.001 | 0.018 | 0.9620 | 0.067 | 0.026 | 0.0109 |
| CCL17 | 0.106 | 0.018 | < 10^-8^ | 0.088 | 0.019 | < 10^-5^ |
| IFN-γ | -0.074 | 0.015 | < 10^-5^ | -0.098 | 0.020 | < 10^-5^ |
| IL10 | -0.001 | 0.017 | 0.9637 | -0.060 | 0.017 | 0.0005 |
| IL2 | -0.098 | 0.020 | < 10^-5^ | -0.124 | 0.021 | < 10^-8^ |
| IL6 | 0.050 | 0.016 | 0.0020 | -0.037 | 0.017 | 0.0347 |
| IL8 | 0.023 | 0.014 | 0.0962 | -0.016 | 0.014 | 0.2592 |
| TNF-α | 0.032 | 0.013 | 0.0127 | -0.044 | 0.011 | 0.0001 |

| Table S7: Biomarkers associated with FEV_1_/FVC | | | | | | | |
| --- | --- | --- | --- | --- | --- | --- | --- |
|  |  | COPDGene |  |  | SPIROMICS | | Combined |
| biomarker | ß | R^2^_(adj)_ | P | ß | R^2^_(adj)_ | P | P |
| Eotaxin | -0.360 | 0.009 | < 10^-5^ | -0.599 | 0.024 | 0.0000 | < 10^-6^ |
| IL6 | -0.208 | 0.013 | < 10^-6^ | -0.376 | 0.025 | 0.0000 | < 10^-6^ |
| R^2^_(adj)_ is the partial amount of additional variance explained by the biomarker in models with clinical covariates | | | | | | | |

| **Table S8**: Amount of variance explained by biomarker and clinical covariate alone and in combination | | | | | | | | | | |
| --- | --- | --- | --- | --- | --- | --- | --- | --- | --- | --- |
|  | | COPDGene | | | | | SPIROMICS | | | |
| Outcome | *Biomarker* | | *Biomarker alone* | *Clinical Covariates* | *Biomarker + clinical covariates* | *Biomarker alone* | | *Clinical Covariates* | *Biomarker + clinical covariates* |  |
| FEV_1_% | *eotaxin* | | 0.007 | NA | NA | 0.039 | | NA | NA |  |
| FEV_1_% | *IL2* | | 0.002 | NA | NA | 0.007 | | NA | NA |  |
| FEV_1_% | *IL6* | | 0.049 | NA | NA | 0.042 | | NA | NA |  |
| FEV_1_% | *IL8* | | 0.002 | NA | NA | 0.005 | | NA | NA |  |
| FEV_1_/FVC | *eotaxin* | | 0.020 | 0.145 | 0.154 | 0.037 | | 0.086 | 0.110 |  |
| FEV_1_/FVC | *IL6* | | 0.022 | 0.145 | 0.158 | 0.032 | | 0.086 | 0.111 |  |
| Decline FEV_1_ | *IL6* | | 0.058 | 0.330 | 0.360 | 0.044 | | 0.262 | 0.297 |  |
| Emphysema Progression | *IL6* | | 0.002 | 0.380 | 0.380 | unavailable | | unavailable | unavailable |  |
| Emphysema Progression | *IL8* | | 0.000 | 0.380 | 0.379 | unavailable | | unavailable | unavailable |  |
| Note that different methods are used to estimate explanation of variance for each statistical model and inter-model estimates should not be made; see methods for details; NA – not applicable since FEV_1_%already contains clinical covariates. | | | | | | | | | |  |

| Table S9: Biomarkers associated with decline in FEV_1_ (ml/yr) for all subjects | | | | | | | |
| --- | --- | --- | --- | --- | --- | --- | --- |
|  |  | COPDGene |  |  | SPIROMICS | | Combined |
| biomarker | ß | R^2^_(mod)_ | P | ß | R^2^_(adj)_ | P | P |
| none |  | 0.330 |  |  | 0.262 |  |  |
| Eotaxin | -0.013 | 0.331 | 0.0528 | 0.004 | 0.277 | 0.8471 | 0.126 |
| IL6 | -0.007 | 0.360 | 0.0263 | 0.021 | 0.297 | 0.1573 | 0.260 |
| R^2^_(mod)_ is the amount of variance explained by the mixed model with clinical covariates | | | | | | | |

| Table S10: Biomarkers associated with decline in FEV_1_ (ml/yr) by COPD or no COPD in COPDGene subjects | | | | | | | |
| --- | --- | --- | --- | --- | --- | --- | --- |
|  | No COPD | | | COPD | | |  |
| biomarker | ß | R^2^_(mod)_ | P | ß | R^2^_(adj)_ | P |  |
| none |  | 0.519 |  |  | 0.258 |  |  |
| Eotaxin | 0.005 | 0.519 | 0.5353 | -0.023 | 0.259 | 0.0199 |  |
| CCL26 | 0.006 | 0.517 | 0.1357 | 0.001 | 0.257 | 0.7354 |  |
| CCL17 | 0.002 | 0.522 | 0.6090 | 0.000 | 0.257 | 0.9441 |  |
| IFN-γ | -0.009 | 0.519 | 0.0497 | 0.001 | 0.257 | 0.8924 |  |
| IL10 | 0.000 | 0.521 | 0.9285 | -0.009 | 0.262 | 0.0861 |  |
| IL2 | 0.001 | 0.513 | 0.6951 | -0.010 | 0.251 | 0.0425 |  |
| IL6 | -0.005 | 0.543 | 0.2963 | -0.007 | 0.271 | 0.2268 |  |
| IL8 | 0.000 | 0.520 | 0.9512 | -0.012 | 0.260 | 0.0619 |  |
| TNF-α | -0.002 | 0.525 | 0.6849 | -0.007 | 0.259 | 0.3259 |  |
| R^2^_(mod)_ is the amount of variance explained by the mixed model with clinical covariates; COPD includes GOLD 1-4; non-COPD all others | | | | | | | |

**Figure S1**: Subtyping of subjects based on airflow obstruction (FEV_1_/FVC) and emphysema severity (LAA% < -950 HU). The vertical line represents the cutoff for COPD (post-bronchodilator FEV_1_/FVC < 0.7). The horizontal line represents the cutoff for emphysema (LAA > 5%). Subjects with chronic bronchitis are shown by red cross and those without a blue circle. The upper panel shows those included in analysis and the lower panels show the whole cohort for COPDGene (left) and SPIROMICS (right).

Whole Cohrot

Analysis subset

SPIROMICS

COPDGene


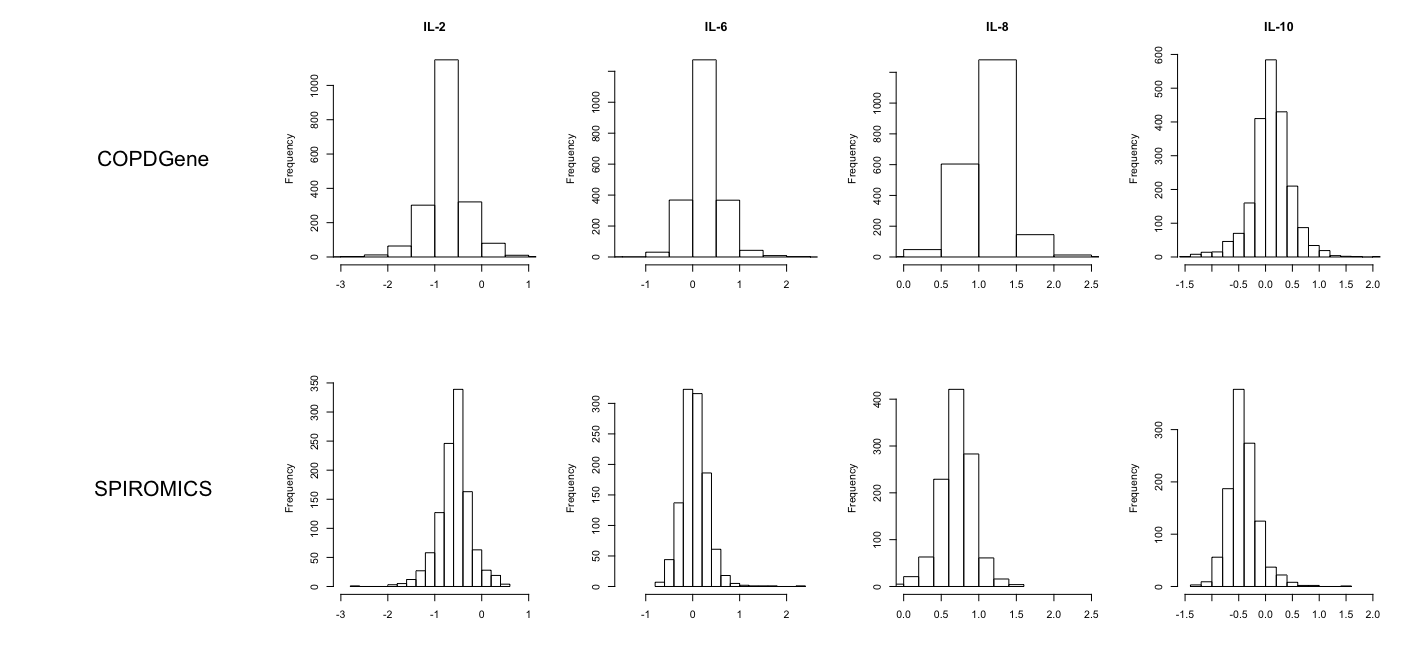


**Figure S2**: Histograms of chemokines and cytokines in COPDGene and SPIROMICS (log10 transformed). Units are pg/ml


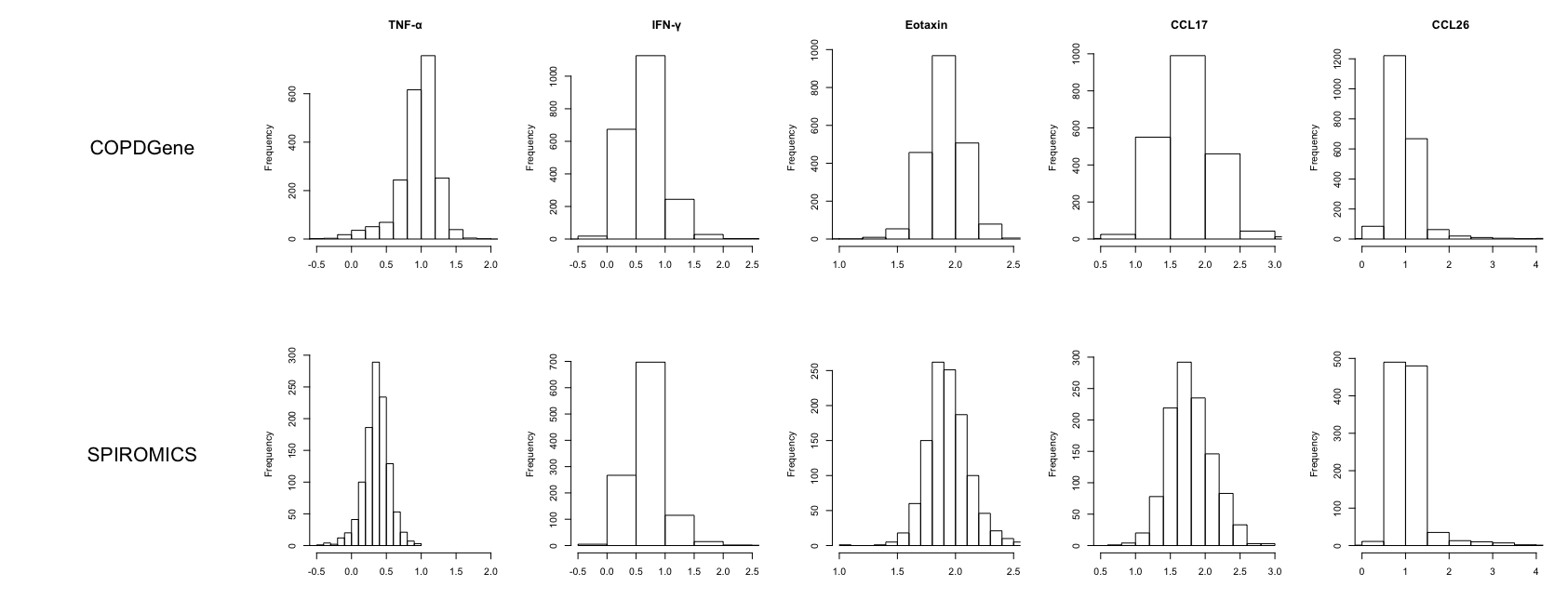


**Figure S3.** Pearson correlations between cytokines/chemokines and cell counts in COPDGene and SPIROMICS. Red squares are positive correlation coefficients and blue negative correlation coefficients with P <0.05. Cell counts were obtained by automated complete blood cell counts. The shading of each cell represents the correlation coefficient as indicated in the legend.


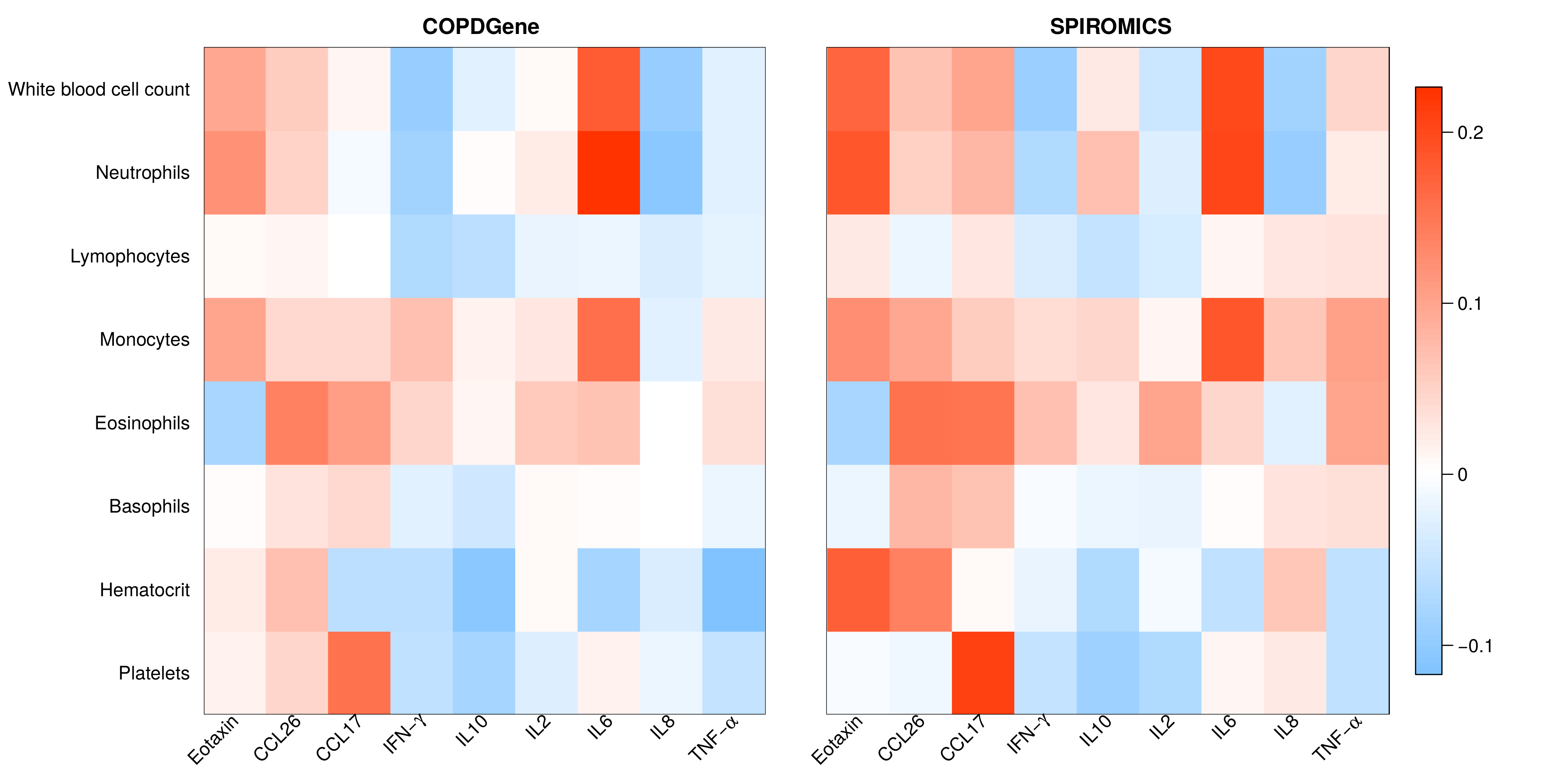


**Figure S4**: Combined Manhattan plots for all 9 biomarker-genotype associations in non-Hispanic White subjects from the COPDGene cohort \. The redline represents genome wide significance level adjusted for multiple testing (P < 10^-9^). Results for all 9 biomarkers are superimposed on the graph. Only one SNP was significantly associated with a biomarker (rs9302690 in CCL17; P = 10^-11^).

CCL17

**Figure S5:** Beeswarm of plasma CCL17 measurements by rs9302690 genotype in the non-Hispanic White subjects in the COPDGene cohort. Subjects with the minor allele had significantly higher levels of CCL17 (P < 10^-11^). Boxplots indicate 25^th^, 50^th^, and 75^th^ percentiles.
