# Supplementary material for: The value of blood cytokines and chemokines in assessing COPD
Source: Respir Res. 2017 Oct 24;18:180. doi: 10.1186/s12931-017-0662-2 (PMC5655820; doi:10.1186/s12931-017-0662-2)
Supplement: Supplementary file 2 — Cross Sectional Associations by Subgroup Heat Map. (PDF 156 kb) [file 12931_2017_662_MOESM2_ESM.pdf]

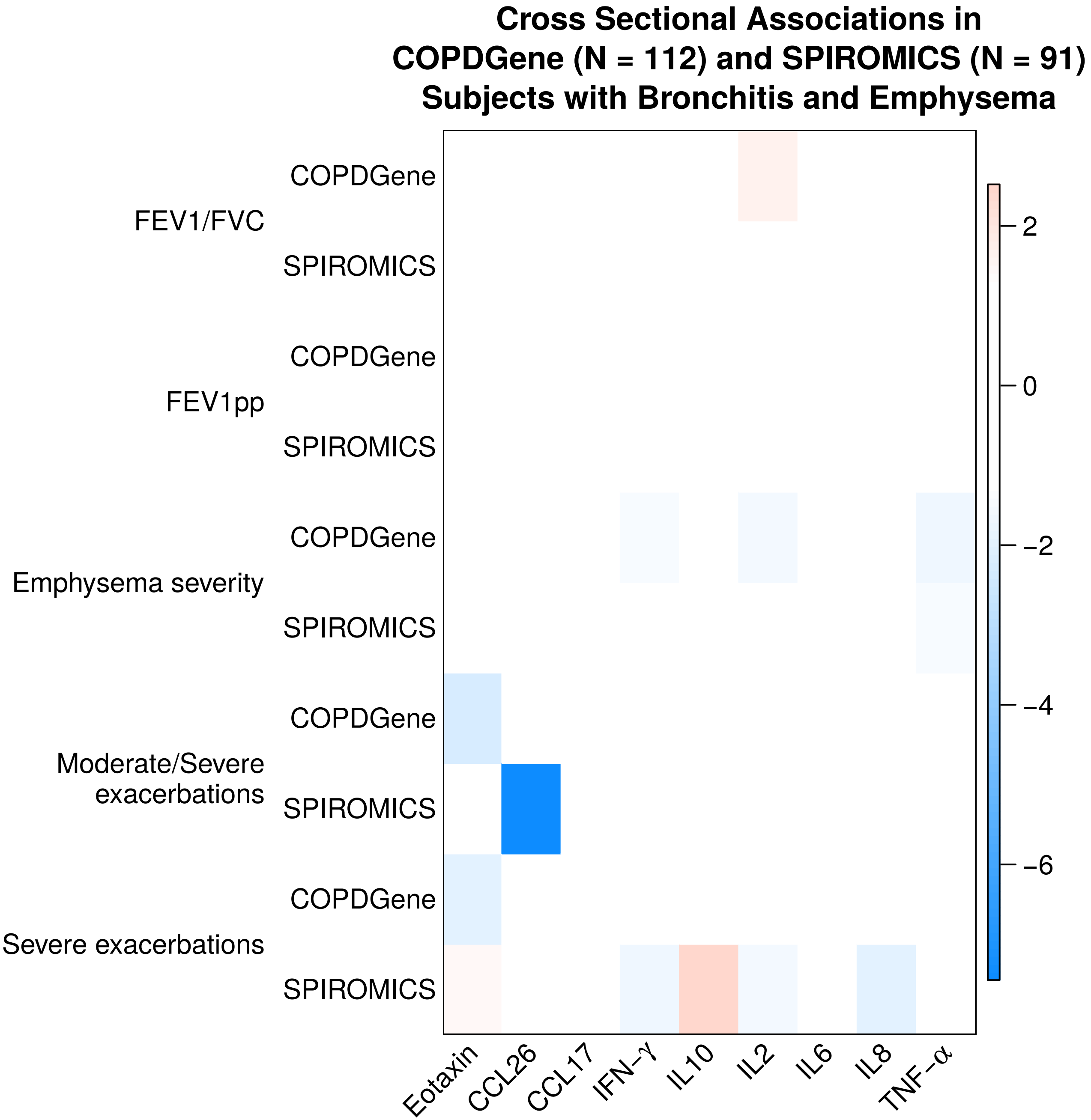
Cross Sectional Associations in
COPDGene (N = 112) and SPIROMICS (N = 91)
Subjects with Bronchitis and Emphysema
FEV1/FVC
FEV1pp
Emphysema severity
Moderate/Severe exacerbations
Severe exacerbations
COPDGene
SPIROMICS
Eotaxin
CCL26
CCL17
IFN−γ
IL10
IL2
IL6
IL8
TNF−α
2
0
−2
−4
−6

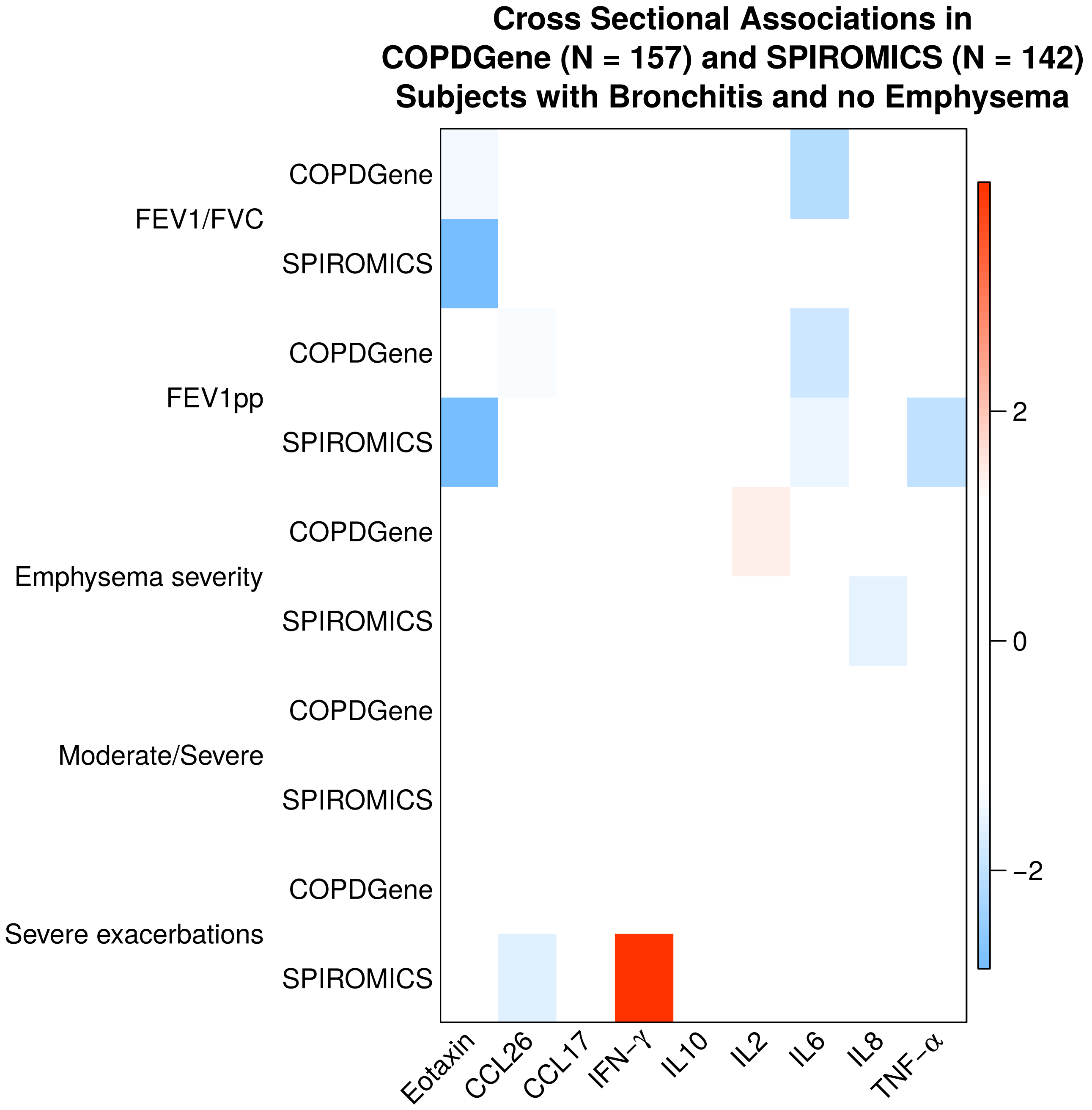
Cross Sectional Associations in
COPDGene (N = 157) and SPIROMICS (N = 142)
Subjects with Bronchitis and no Emphysema
FEV1/FVC
COPDGene
SPIROMICS
FEV1pp
COPDGene
SPIROMICS
Emphysema severity
COPDGene
SPIROMICS
Moderate/Severe
COPDGene
SPIROMICS
Severe exacerbations
COPDGene
SPIROMICS
Eotaxin
CCL26
CCL17
IFN−γ
IL10
IL2
IL6
IL8
TNF−α
2
0
−2

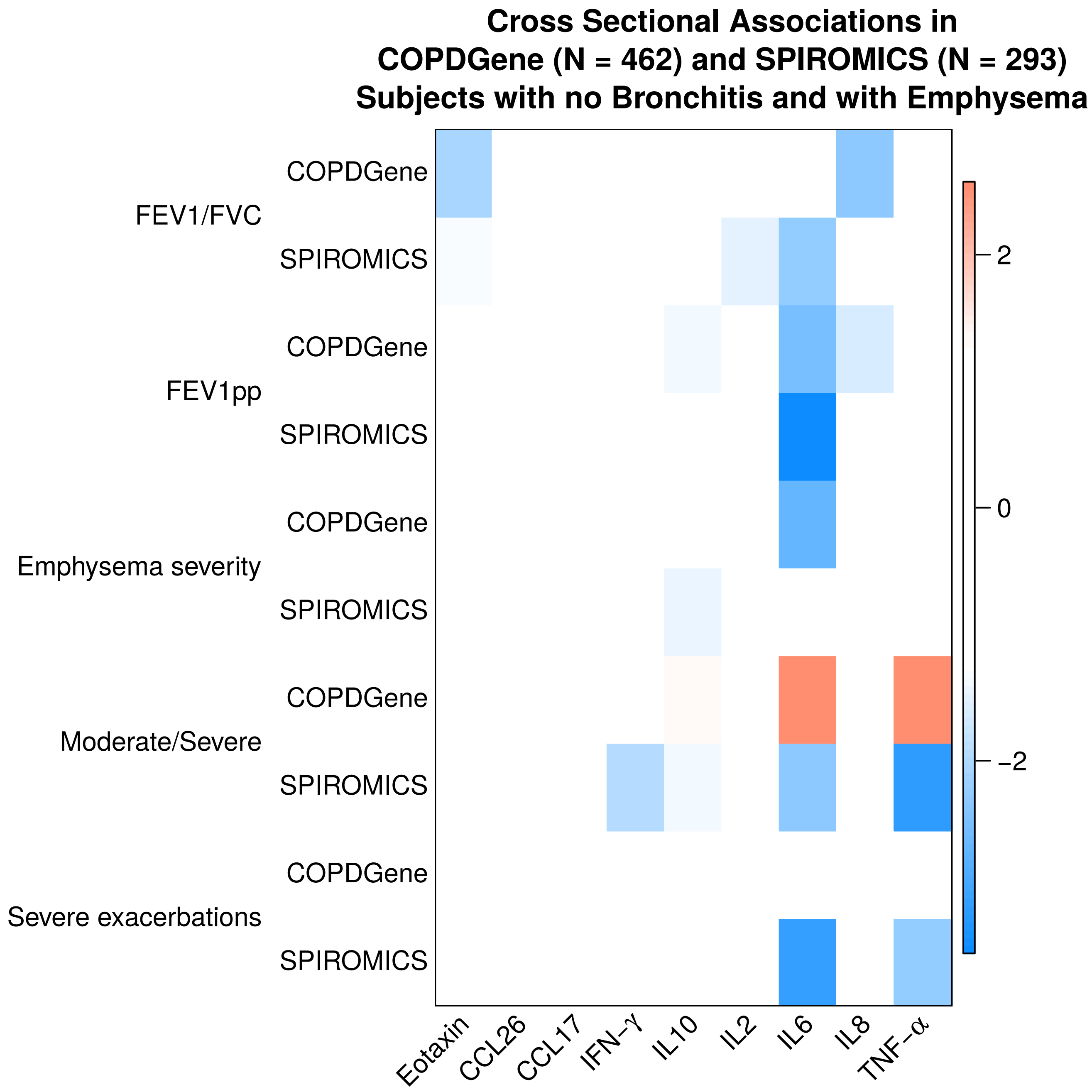
Cross Sectional Associations in
COPDGene (N = 462) and SPIROMICS (N = 293)
Subjects with no Bronchitis and with Emphysema
FEV1/FVC
FEV1pp
Emphysema severity
Moderate/Severe
Severe exacerbations
COPDGene
SPIROMICS
Eotaxin
CCL26
CCL17
IFN−γ
IL10
IL2
IL6
IL8
TNF−α
2
0
−2

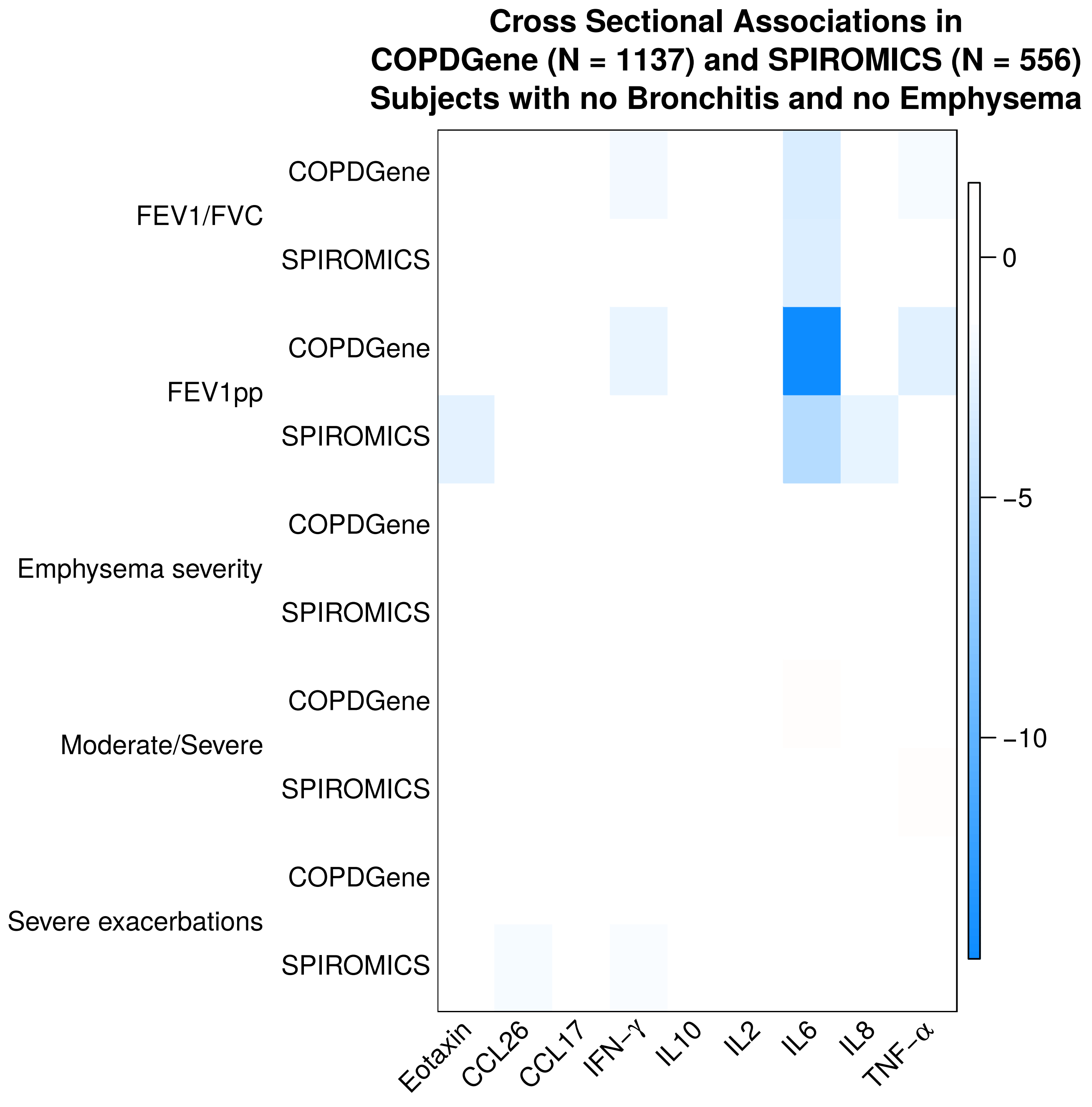

Cross Sectional Associations in
COPDGene (N = 1137) and SPIROMICS (N = 556)
Subjects with no Bronchitis and no Emphysema
FEV1/FVC
COPDGene
SPIROMICS
FEV1pp
COPDGene
SPIROMICS
Emphysema severity
COPDGene
SPIROMICS
Moderate/Severe
COPDGene
SPIROMICS
Severe exacerbations
COPDGene
SPIROMICS
Eotaxin
CCL26
CCL17
IFN−γ
IL10
IL2
IL6
IL8
TNF−α
0
−5
−10

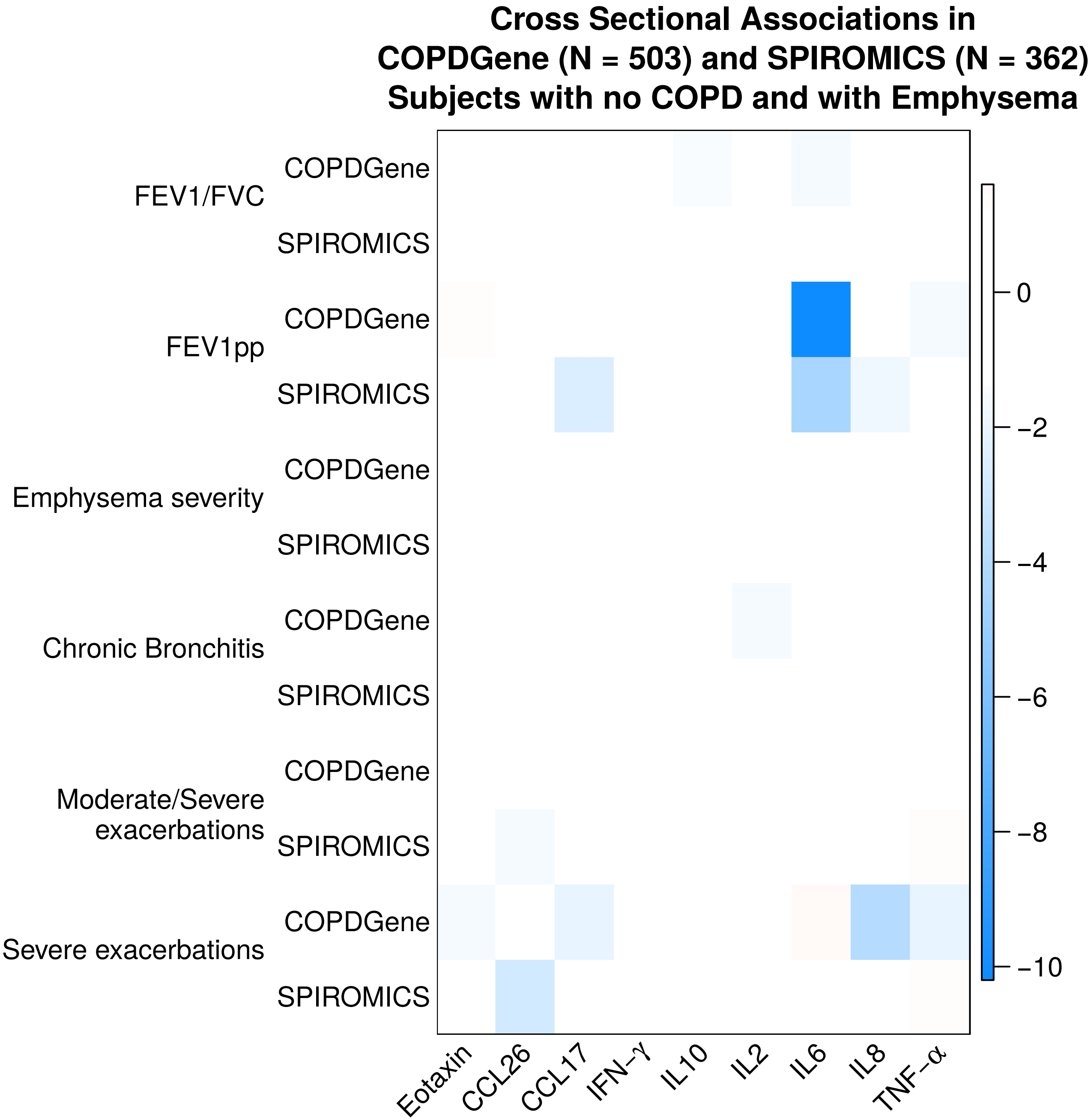

Cross Sectional Associations in
COPDGene (N = 503) and SPIROMICS (N = 362)
Subjects with no COPD and with Emphysema
FEV1/FVC
FEV1pp
Emphysema severity
Chronic Bronchitis
Moderate/Severe exacerbations
Severe exacerbations
COPDGene
SPIROMICS
Eotaxin
CCL26
CCL17
IFN−γ
IL10
IL2
IL6
IL8
TNF−α
0
−2
−4
−6
−8
−10

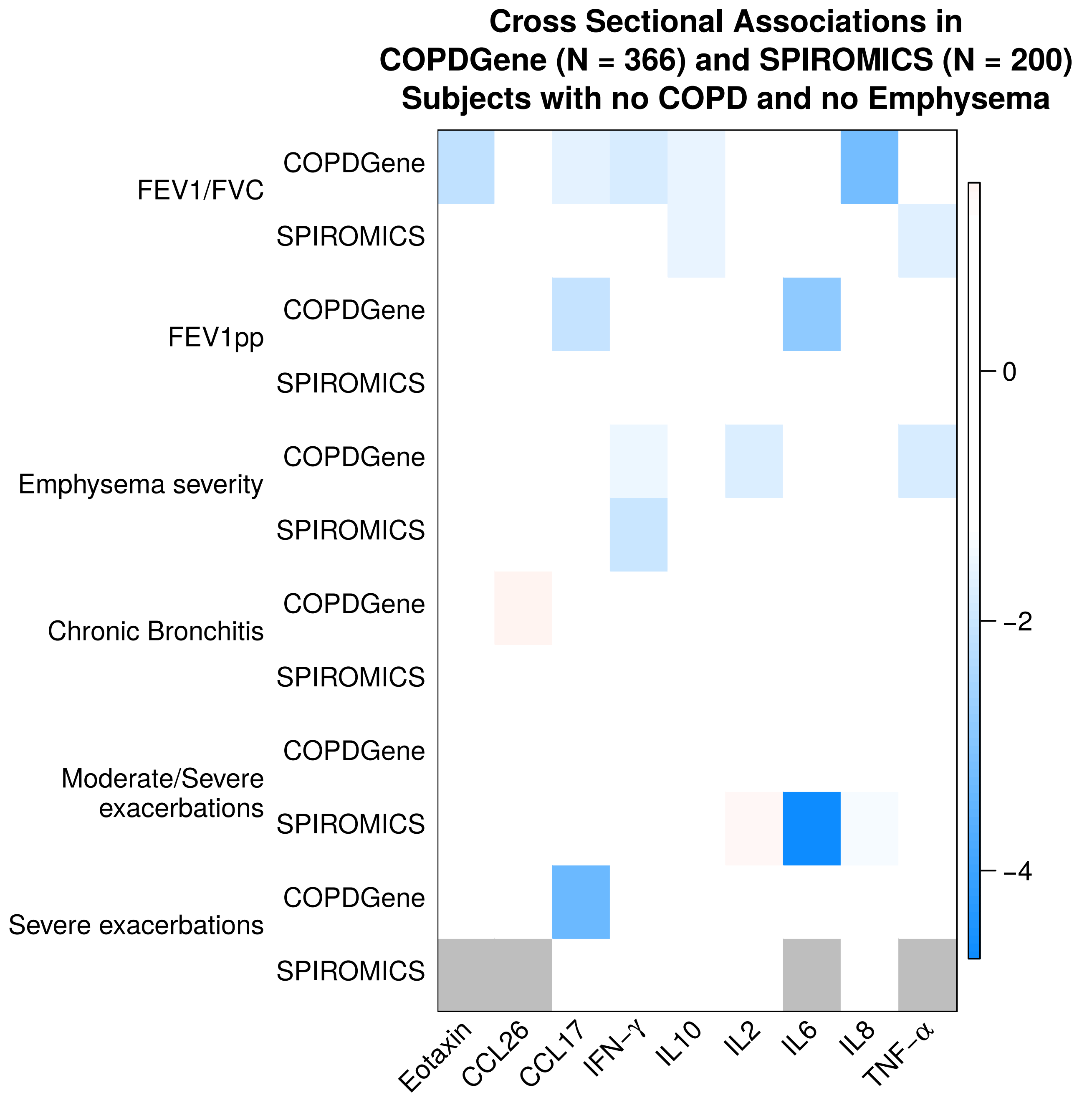
Cross Sectional Associations in
COPDGene (N = 366) and SPIROMICS (N = 200)
Subjects with no COPD and no Emphysema
FEV1/FVC
FEV1pp
Emphysema severity
Chronic Bronchitis
Moderate/Severe exacerbations
Severe exacerbations
COPDGene
SPIROMICS
Eotaxin
CCL26
CCL17
IFN−γ
IL10
IL2
IL6
IL8
TNF−α
0
−2
−4
