# Supplementary material for: The value of blood cytokines and chemokines in assessing COPD
Source: Respir Res. 2017 Oct 24;18:180. doi: 10.1186/s12931-017-0662-2 (PMC5655820; doi:10.1186/s12931-017-0662-2)

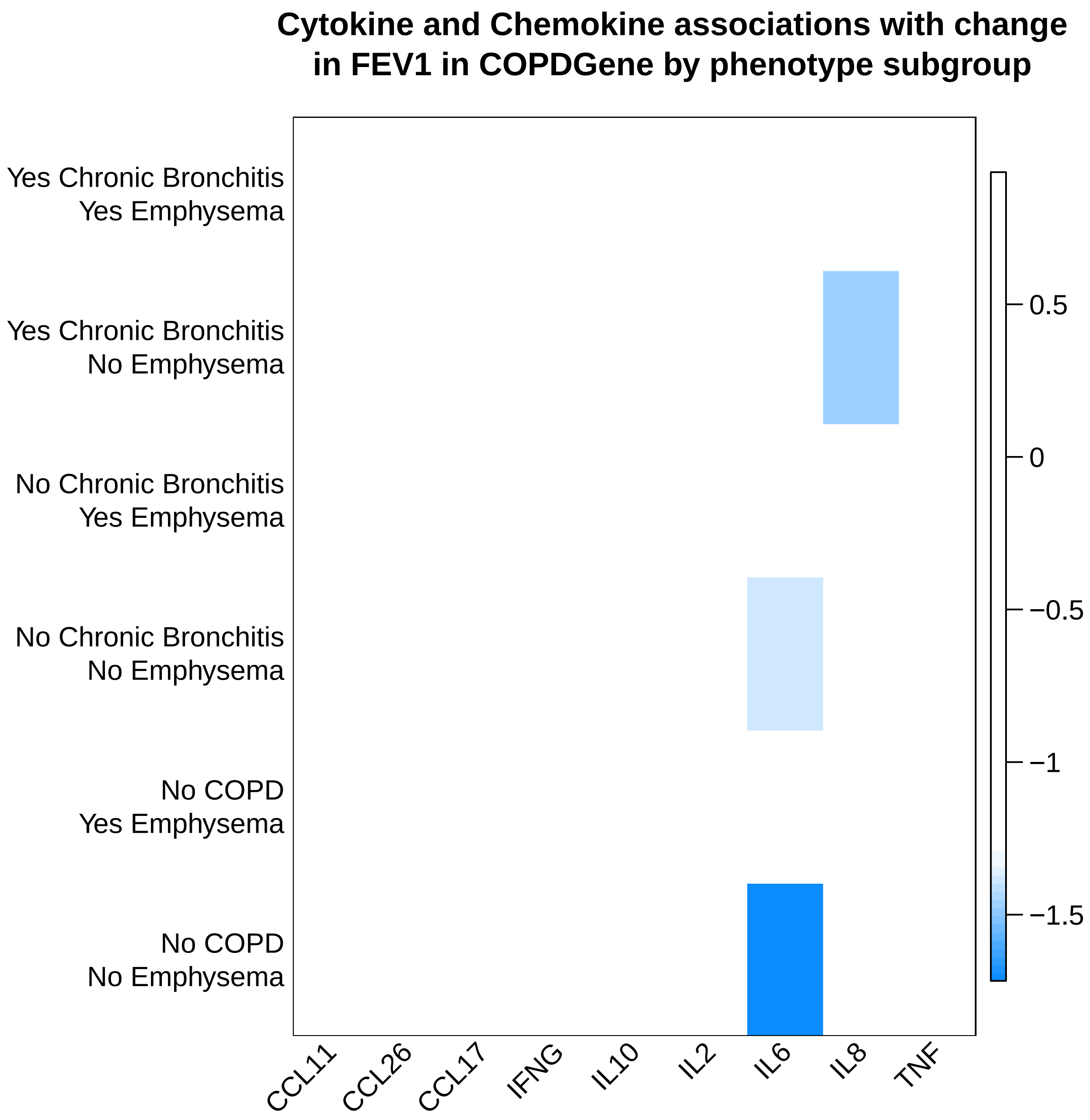
Cytokine and Chemokine associations with change in FEV1 in COPDGene by phenotype subgroup
Yes Chronic Bronchitis
Yes Emphysema
Yes Chronic Bronchitis
No Emphysema
No Chronic Bronchitis
Yes Emphysema
No Chronic Bronchitis
No Emphysema
No COPD
Yes Emphysema
No COPD
No Emphysema
CCL11
CCL26
CCL17
IFNG
IL10
IL2
IL6
IL8
TNF
0.5
0
−0.5
−1
−1.5

Cytokine and Chemokine associations with progression of Emphysema in COPDGene by phenotype subgroup

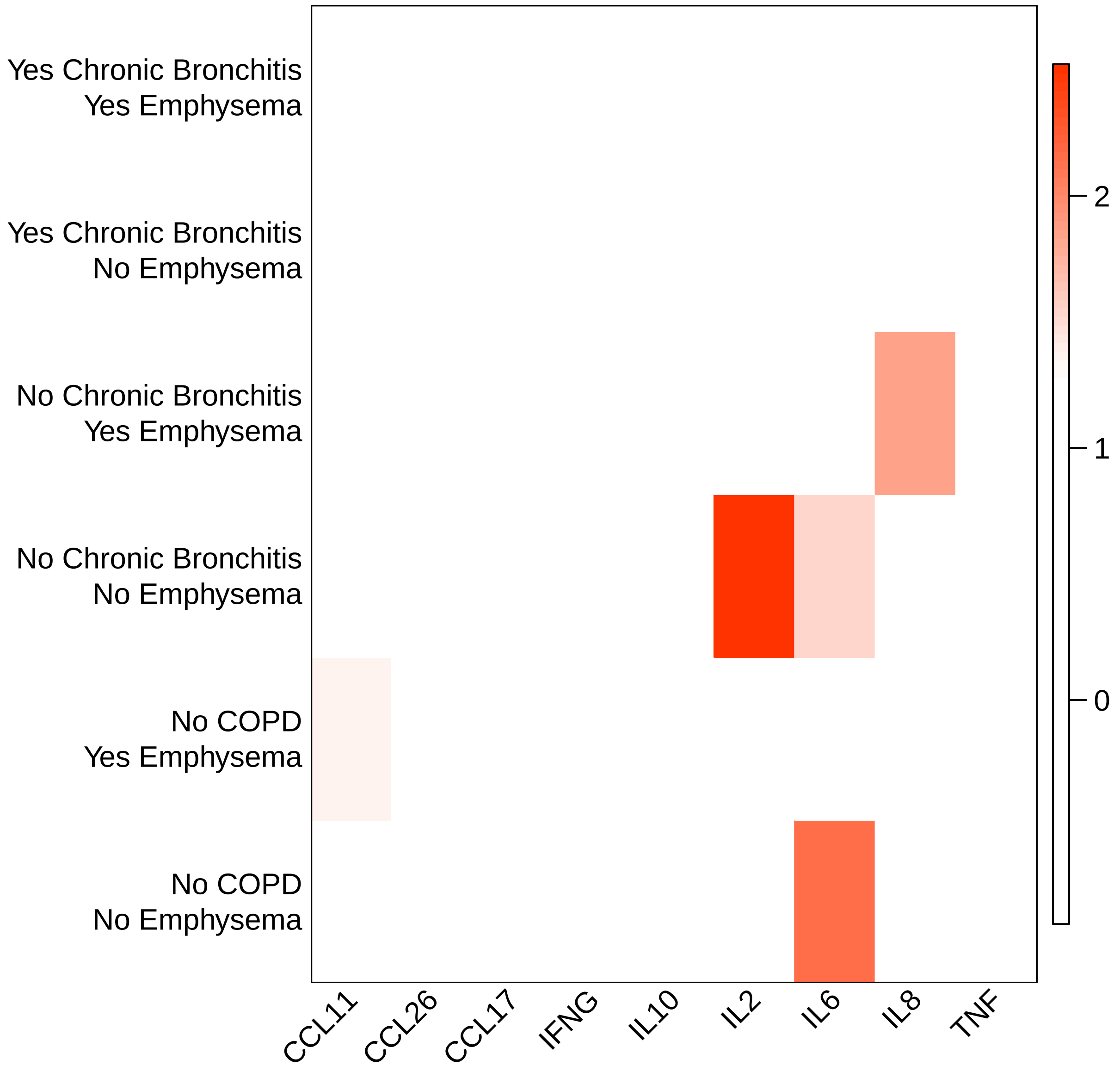

Supplement: Supplementary file 3 — Associations with Disease Progression by Subgroup Heatmap. (PDF 7 kb) [file 12931_2017_662_MOESM3_ESM.pdf]
